# Urine Extracellular Vesicle miRNA Changes Induced by Vicadrostat with/Without Empagliflozin in Patients with Chronic Kidney Disease

**DOI:** 10.3390/ijms262210810

**Published:** 2025-11-07

**Authors:** Denis Delic, Isabella Gashaw, Ileana Duran-Fernandez, Lisa Cronin, Sibylle J. Hauske, Peter Rossing, Katherine R. Tuttle

**Affiliations:** 1 Boehringer Ingelheim Pharma GmbH & Co. KG, 88400 Biberach, Germany; 2 Fifth Department of Medicine (Nephrology/Endocrinology/Rheumatology), University Medical Centre Mannheim, University of Heidelberg, 68167 Heidelberg, Germany; sibylle.hauske@boehringer-ingelheim.com; 3 Boehringer Ingelheim Pharma GmbH & Co. KG, 55216 Ingelheim am Rhein, Germany; isabella.gashaw@boehringer-ingelheim.com; 4 Staburo GmbH, 81549 Munich, Germany; ileana.duran_fernandez.ext@boehringer-ingelheim.com; 5 Boehringer Ingelheim Pharmaceuticals, Ridgefield, CT 06877, USA; lisa.cronin@boehringer-ingelheim.com; 6 Boehringer Ingelheim International GmbH, 55216 Ingelheim am Rhein, Germany; 7 Steno Diabetes Center Copenhagen, 2730 Herlev, Denmark; peter.rossing@regionh.dk; 8 Department of Clinical Medicine, University of Copenhagen, 2200 Copenhagen, Denmark; 9 Providence Inland Northwest Health, Spokane, WA 99201, USA; katherine.tuttle@providence.org; 10 Nephrology Division, Kidney Research Institute, University of Washington, Seattle, WA 98195, USA

**Keywords:** aldosterone synthase inhibition, SGLT2 inhibition, albuminuria, biomarkers, diabetes

## Abstract

Vicadrostat, a selective aldosterone synthase inhibitor, reduced albuminuria with concurrent renin–angiotensin system inhibition and empagliflozin, suggesting additive efficacy for chronic kidney disease (CKD) treatment. Specific urinary extracellular vesicle microRNAs (uEV miRNAs) may reflect key mechanisms of kidney injury. We investigated how vicadrostat alone or with empagliflozin affected uEV miRNA expression in study participants. Small RNA sequencing was conducted on uEV miRNAs from 435 participants with CKD who completed 14 weeks treatment in the phase II trial of vicadrostat given with or without empagliflozin. Differentially expressed uEV miRNAs in participants with ≥30% UACR (urine albumin–creatinine ratio) reduction treated with 10 or 20 mg vicadrostat were pooled and evaluated with or without empagliflozin. Changes in miRNA-142-5p correlated significantly with changes in UACR in participants treated with vicadrostat alone, whereas changes in expression of eight additional uEV miRNAs (miR-192-5p, miR-194-5p, miR-6882-5p, miR-27a-5p, miR-381-3p, miR-192-3p, miR-513a-5p, and miR-199b-3p) correlated with ≥30% UACR improvements in patients treated with vicadrostat plus empagliflozin. Cellular deconvolution revealed that these miRNAs were expressed in various kidney cell types. Vicadrostat plus empagliflozin altered uEV miRNAs involved in immunomodulatory and fibrotic pathways irrespective of participant diabetes status. Regulation of miRNAs may provide insights into synergistic mechanisms of vicadrostat and empagliflozin in CKD treatment.

## 1. Introduction

The global burden of chronic kidney disease (CKD) is substantial and growing: approximately 10% of adults worldwide have CKD, resulting in 1.2 million deaths each year [1,2,3]. By 2040, CKD is estimated to become the fifth leading cause of death globally—one of the largest projected increases in any major cause of death [4,5]. First-line treatment for CKD includes renin–angiotensin system (RAS) inhibition, in the form of angiotensin converting enzyme (ACE) inhibitors or angiotensin receptor blockers (ARBs), and sodium-glucose co-transporter 2 inhibitors (SGLT2i) [6]. Risk-based therapy for people with CKD and type 2 diabetes (T2D) who have residual albuminuria includes a non-steroidal mineralocorticoid receptor antagonist (MRA). Although ACE inhibitors, ARBs, and non-steroidal MRAs provide benefit for CKD, they do not fully block the effects of aldosterone and increase the risk of hyperkalemia [7,8,9]. Vicadrostat is a potent, highly selective aldosterone synthase inhibitor in clinical development in combination with the SGLT2i empagliflozin (VicaEmpa) for treatment of CKD and heart failure. In a phase 2, randomized, placebo-controlled clinical trial, vicadrostat dose-dependently reduced albuminuria with concurrent renin–angiotensin system inhibition and empagliflozin, suggesting additive efficacy [10].

The underlying molecular mechanisms remain to be elucidated for the effects of vicadrostat in CKD. Urine albumin–creatine ratio (UACR) and estimated glomerular filtration rate (eGFR) are commonly used biomarkers in chronic kidney disease trials but not ideal for molecular differentiation between treatments during disease progression. In recent years, studies have underlined the importance of exosomes as liquid biopsies and a novel source of biomarkers in kidney diseases [11]. Furthermore, miRNAs exhibit superior stability in degraded RNA samples, which makes them more suitable biomarkers [12].

MiRNAs are small non-coding RNA molecules that regulate gene expression, and have important roles in CKD [13,14]. In particular, miRNAs promote tubulointerstitial and glomerular fibrosis [13]. Urine exosomes contain miRNAs packaged in extracellular vesicles that are secreted in large quantities from all nephron segments [15]. Urinary exosomal miRNA content is altered in patients with focal segmental glomerulosclerosis [16,17] and in patients with type 1 or type 2 diabetes and CKD [18,19,20]. Notably, human urinary extracellular vesicle (uEV) miRNA patterns may also be used to identify cellular targets of drug effects [21].

In the present study, we investigated effects of vicadrostat plus empagliflozin or vicadrostat alone on uEV miRNA expression to evaluate their potential mechanistic effects in CKD.

## 2. Results

### 2.1. Effects of Vicadrostat_high_, Empagliflozin, and Vicadrostat_high_ Plus Empagliflozin Resulted in Unique uEV miRNA Profiles

The baseline characteristics of the study population (Table 1) were similar to the overall trial population [22].

**Figure 1:**
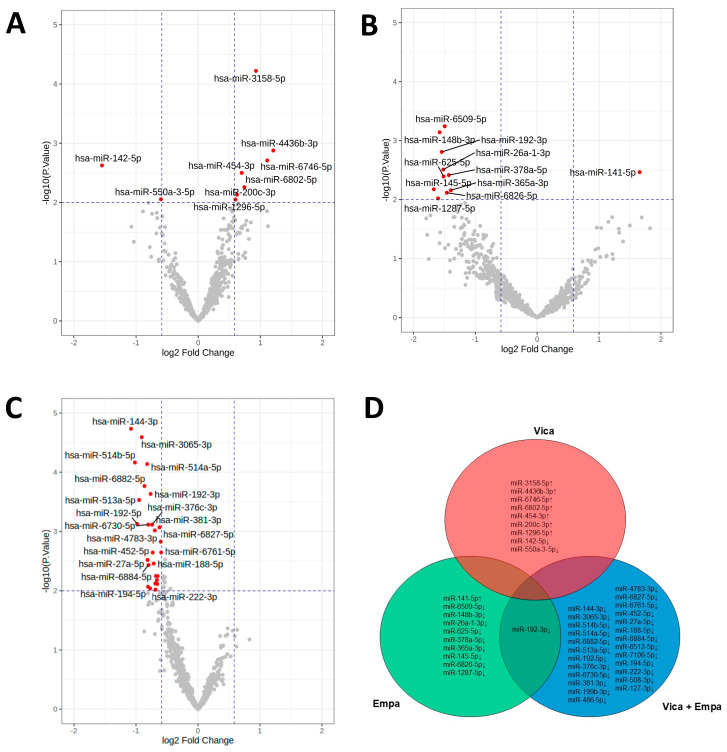
Effects of vicadrostat, empagliflozin, and vicadrostat plus empagliflozin on uEV expression. Treatment-mediated changes in miRNAs in patients with CKD receiving Vica_high_ alone (*n* = 49) (**A**), empa alone (*n* = 11) (**B**), or Vica_high_ plus empa (*n* = 63) (**C**) who showed ≥30% reduction in UACR. (**D**) Venn diagram illustrating unique and overlapping, significantly regulated miRNA species after treatment with therapeutic doses of vica (red) or empa (green) alone or in combination (vica plus empa) (blue). Arrows indicate direction of deregulation. Vertical and horizontal dotted lines represent the fold-change and *p*-value cut-offs. Differentially expressed miRNAs fulfilling the following criteria: *p*-value ≤ 0.01, |log2 fold-change| ≥ 0.585 are highlighted by red dots. Vica_high_ = Vica 10 mg + Vica 20 mg; Vica, vicadrostat; Empa, empagliflozin.

To investigate a potential molecular mechanism for the effect of vicadrostat treatment in CKD, uEV miRNA profiling was performed. Percentage change in UACR from baseline to the end of treatment at week 14 was similar in participants who received the high doses (10 or 20 mg) vicadrostat [10]. A subgroup analysis was performed on patients with CKD receiving high doses (10 or 20 mg) of vicadrostat (vicadrostat_high_) who showed ≥30% reduction in UACR compared to baseline. An exploratory analysis of 724 miRNAs present in uEV revealed that miRNAs were modulated (≥1.5 fold; *p* ≤ 0.01) by the vicadrostat_high_ treatment in this subgroup; the expression of the seven miRNAs miR-3158-5p, miR-4436b-3p, miR-6746-5p, miR-454-3p, miR-6802-5p, miR-200c-3p, and miR-1296-5p increased, whereas the expression of the two miRNAs miR-550a-3-5p and miR-142-5p decreased in patients with CKD receiving vicadrostat_high_ who showed a reduction of ≥30% in UACR (Figure 1A). Participants who were treated with empagliflozin alone and experienced ≥30% UACR reduction are characterized by an increased uEV level of miR-141-5p and decreased levels of miR-6509-5p, miR-148b-3p, miR-192-3p, miR-26a-1-3p, miR-625-5p, miR-378a-5p, miR-145-5p, miR-365a-3p, miR-6826-5p, and miR-1287-5p (Figure 1B). Participants with ≥30% UACR reduction following vicadrostat_high_ plus empagliflozin treatment experienced a decreased expression of 26 miRNAs (Figure 1C). Interestingly, only a decreased level of miR-192-3p overlapped between empagliflozin mono treatment and treatment in combination with vicadrostat (Figure 1D).

### 2.2. Changes in uEV miRNA Expression Profiles Associated with Albuminuria

**Figure 2:**
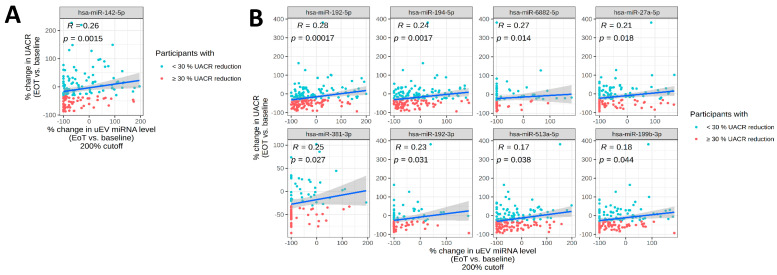
Correlation between changes in uEV miRNAs and UACR. (**A**) Significant correlation between Vica_high_-derived changes in uEV miRNAs and changes in UACR (in all participants without empa in run-in period). (**B**) Significant correlation between Vica_high_ + Empa-derived changes in uEV miRNAs and changes in UACR (in all participants with empa in run-in period). Gray shaded areas indicate 95% confidence interval. EoT = End of treatment (week 14).

Due to small numbers of participants receiving empagliflozin-only treatment, the following analyses are focused on vicadrostat monotherapy and vicadrostat plus empagliflozin combination therapy. The correlation analysis revealed that the % changes in uEV miR-142-5p expression significantly correlated with % changes in UACR (*r*^2^ = 0.26; *p* = 0.0015) (Figure 2A). The combined therapy with vicadrostat and empagliflozin resulted in significant correlations between expression changes and UACR for the following miRNAs: miR-192-5p (*r*^2^ = 0.28; *p* < 0.001), miR-194-5p (*r*^2^ = 0.24; *p* = 0.0017), miR-6882-5p (*r*^2^ = 0.27; *p* = 0.014), miR-27a-5p (*r*^2^ = 0.21; *p* = 0.018), miR-381-3p (*r*^2^ = 0.25; *p* = 0.027), miR-192-3p (*r*^2^ = 0.23; *p* = 0.031), miR-513a-5p (*r*^2^ = 0.17; *p* = 0.038), and miR-199b-3p (*r*^2^ = 0.18; *p* = 0.044) (Figure 2B).

The differential expression and correlations with UACR of the outcome-related uEV miRNAs are summarized in Table 2.

**Figure 3:**
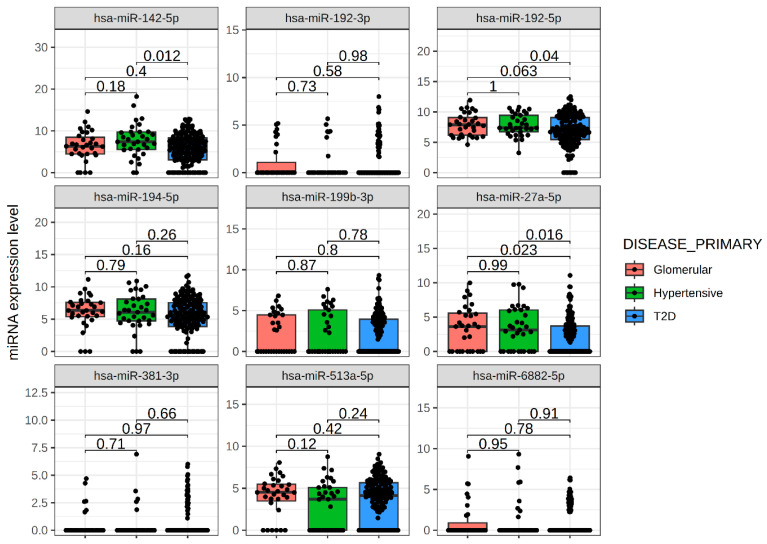
Analysis of differentially expressed uEV miRNAs based on the CKD diagnosis among participants receiving the placebo during the run-in phase. Glomerular: glomerular disease (FSGS, MN, AS) (*n* = 31); Hypertensive: patients with hypertension-associated CKD (*n* = 33); T2D: type 2 diabetes-associated CKD (*n* = 141). P-values indicate level of significance for group comparisons. miRNA expression levels are given in normalized log2 cpm + 1. Values are presented in boxplots (median with interquartile range (IQR)).

Changes in uEV miRNA expression in response to vicadrostat_high_ treatment were not apparent in participants who showed <30% reduction in UACR compared to baseline (Table S1) or in participants treated with placebo or 3 mg vicadrostat with or without empagliflozin who showed ≥30% UACR reduction (Table S2). Alterations of uEV miRNA expression profiles were present irrespective of diabetes status (Table S3). Participants were also categorized by their CKD diagnosis to assess the expression of vicadrostat-regulated miRNAs (Figure 3). Baseline expression of the miRNAs was similar across diagnoses. Strong expression levels in uEV were identified for miR-142-5p, miR-192-5p and miR-194-5p, moderate expression of miR-27a-5p, miR-199b-3p, and miR-513a-5p, as well as a weak expression of miR-192-3p, miR-381-5p, and miR-6882-5p.

### 2.3. Sustained Effects of Vicadrostat on uEV miRNAs Four Weeks Post-Treatment

**Figure 4:**
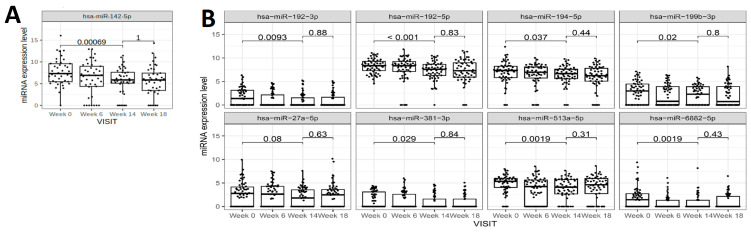
Effects of vicadrostat alone (**A**) and empagliflozin combination therapy (**B**) on uEV miRNAs at 4 weeks after treatment completion in participants with ≥30% UACR reduction. P-values indicate level of significance for group comparisons. miRNA expression levels are given in normalized log2 cpm + 1. Values are presented in boxplots (median with interquartile range (IQR)).

The changes in the pre-selected uEV miRNAs were measured at two additional time-points, namely week 6 and a follow-up visit 4 weeks after discontinuation of the study treatment (Figure 4). The changes in uEV expression profiles were not apparent after week 6 of treatment but sustained after 4 weeks of completion of 14 weeks of treatment with vicadrostat_high_ (Figure 4).

### 2.4. Functional Assessments of Treatment Effects

Established functions of the uEV miRNAs according to kidney diseases are summarized in Table 3.

**Figure 5:**
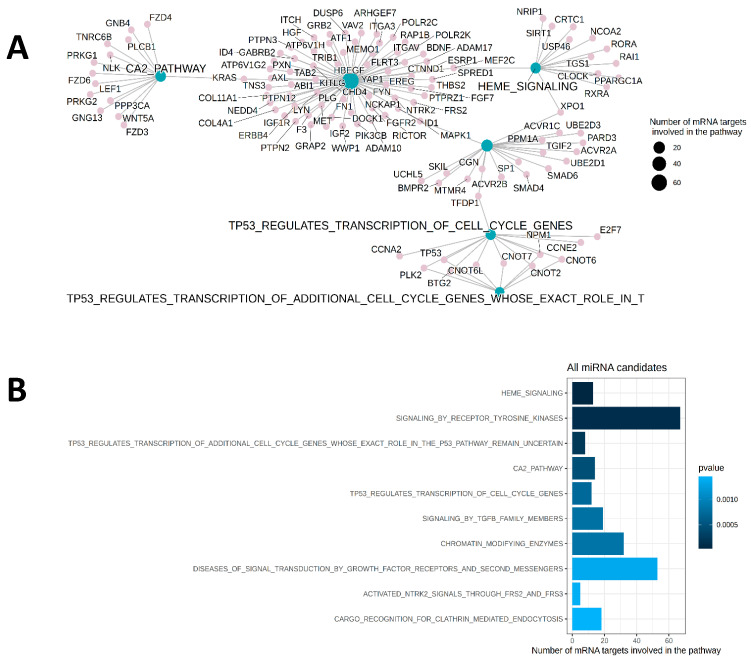
Pathway overrepresentation analysis. (**A**): Network analysis of significantly enriched pathways regulated by the eight identified miRNAs and respective messenger RNA (mRNA) targets involved in the regulation. Size of the blue dots reflects number of mRNA targets (pink) in the pathway (blue). (**B**) Significant enrichment of the top 10 pathways. P-value range is highlighted by the blue scale.

A pathway enrichment analysis with the predicted targetome of the miRNA candidates revealed that pathways such as TGF-beta signaling, heme signaling, and calcium signaling are significantly enriched (Figure 5A) with several direct target mRNAs that are involved in the regulation of the respective signaling pathways (Figure 5B).

**Figure 6:**
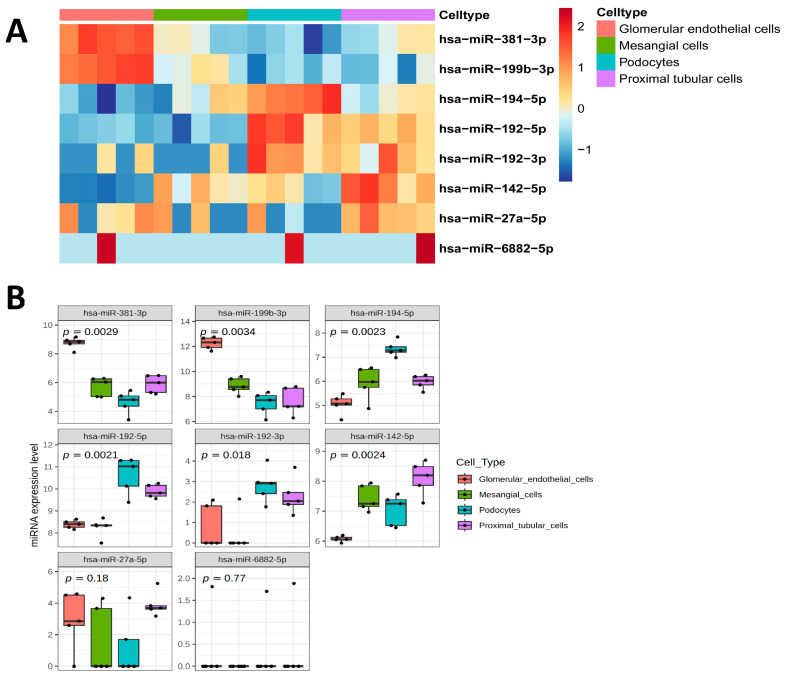
Enriched expression profiles in various kidney cell type-derived EVs. (**A**) Heatmap illustrating expression profiles of identified uEV miRNAs across EVs derived from glomerular endothelial cells, mesangial cells, podocytes, and proximal tubular cells. Expression levels were z-score normalized. (**B**) Box plots show significant enrichment of identified uEV miRNAs across EVs derived from glomerular endothelial cells, mesangial cells, podocytes, and proximal tubular cells. Kruskal–Wallis test *p* values are highlighted at the top of the graphs. miRNA expression levels are given in normalized log2 cpm + 1. *n* = 5/cell type.

Using publicly available data for extracellular vesicle miRNAs obtained from human conditionally immortalized podocytes, glomerular endothelial cells, mesangial cells, and proximal tubular cells, we identified that miR-142-5p showed significantly higher expression in EVs that are reported to be derived from proximal tubular cells, mesangial cells, and podocytes compared to glomerular endothelial cells (Figure 6A,B). miR-192-5p and miR-194-5p are significantly enriched in podocytes and miR-381-3p and miR-199b-3p in glomerular endothelial cells, whereas miR-192-3p and miR-27a-5p are expressed in various kidney cell types (Figure 6B). miR-513a-5p and miR-6882-5p have not been linked previously to EVs of the analyzed kidney cells (Figure 6A,B).

### 2.5. Correlations of uEV miRNA Expression with UACR and eGFR

The changes in uEV miRNA expression levels after vicadrostat_high_ alone or vicadrostat_high_ plus empagliflozin treatment were not significantly correlated with baseline UACR and eGFR (Table S4). Conversely, baseline uEV miRNA levels did not associate with changes in UACR or eGFR (Table S5).

## 3. Discussion

Here we present unique pharmacological effects of three different treatments on uEV miRNAs in a population with CKD. We were able to differentiate potential mechanisms of the individual treatments and provide evidence for the additive effects of vicadrostat plus empagliflozin in CKD based on more pronounced effects on alterations in uEV miRNA levels. The trial design allowed for a direct comparison of the mechanistic effects of vicadrostat alone versus vicadrostat plus empagliflozin. This study focused on changes in miRNAs in patients who experienced more than a 30% reduction in UACR. From the regulated biomarkers, there was one miRNA (miR-142-5p) with a significant relationship to UACR changes on vicadrostat treatment, while eight miRNAs showed correlation to changes in UACR in patients on vicadrostat plus empagliflozin. This observation supports an overall benefit of combined treatment. Additionally, the changes in miRNA profiles were long-lasting, persisting 4 weeks after treatment, suggesting a sustained impact on pathogenic mechanisms like fibrosis and inflammation.

In general, miRNAs are protected from degradation through encapsulation in microvesicles such as exosomes. Urine is rich in exosomes, which are secreted by cells from all nephron segments. Due to mechanical and charge barriers in the glomerulus, circulating microvesicles from serum cannot cross the nephron, suggesting that urinary exosomes originate primarily from kidney cells [21]. High expression levels of targeted miRNAs are present in various kidney cells, suggesting that vicadrostat plus empagliflozin treatment may affect different cell types, and as such, could offer a therapeutic option for diverse CKD etiologies. This is further supported by similar baseline expression patterns of targeted miRNAs across different CKD subgroups including diabetes, hypertension, and glomerular diseases.

Urinary exosomal miRNAs have been considered as potential biomarkers for disease activity in CKD, reflecting specific characteristics of kidney cells. Although their diagnostic and prognostic properties have been recently reported [36], there is limited information on their utility for monitoring treatment. All treatments regulated miR-192-3p, a marker associated with diabetic kidney disease [36], and that has shown high diagnostic accuracy, with 89% sensitivity and specificity [37]. Empagliflozin, vicadrostat, and vicadrostat plus empagliflozin treatments, individually and combined, effectively reduce albuminuria and regulate miR-192-3p independently of diabetes status in the present study. Mechanistically, miR-192 contributes to kidney disease by forming a TGF-β-induced positive feedback loop with p53, suppressing *Zeb2*, and promoting kidney fibrosis, while its inhibition or deletion reduces these pathological features [38]. In a previous preclinical study, empagliflozin treatment significantly improved kidney function and reduced interstitial fibrosis in 5/6 nephrectomy rats [39]. scRNA-seq revealed that empagliflozin modulated the TGF-β signaling pathway, inhibited intercellular communication, and reduced the expression of fibrotic genes such as *Col4a1* or *Fn1* [40] that are associated with miR-192-mediated TGF-beta/SMAD3-driven renal fibrosis [41]. Moreover, treatment with the locked nucleic acid-anti-miRNA-192 reduced kidney fibrosis and proteinuria in diabetic mice [41]. Treatment with vicadrostat plus empagliflozin also resulted in decreased levels of the pro-fibrotic miR-194-5p. Urinary exosomal miR-194-5p was upregulated >3-fold compared to controls in children with nephrotic syndrome and correlated with the degree of proteinuria [42]. The levels of miR-194-5p decreased in parallel with proteinuria reduction when children were treated by immunosuppression [42].

Immunomodulatory effects were also apparent by uEV miRNAs with vicadrostat plus empagliflozin treatment, in addition to the vicadrostat effects associated with fibrosis. A decreased level of the oncogenic miRNA-381-3p, which is a dual suppressor of TNF-induced apoptosis and necroptosis that promotes the proliferation of kidney cancer cells, was observed [29]. Vicadrostat plus empagliflozin treatment also reduced the uEV miR-27a-5p level, which is involved in the regulation of NF-kB signaling [28].

This study has several notable limitations. The clinical trial was conducted at 204 sites in 29 countries [22]. While urine sampling adhered to a standardized protocol, variability in procedural conduct is possible [43]. Furthermore, novel methodologies are required for absolute quantification of uEV miRNA levels. Nevertheless, the informative results obtained from uEV miRNA profiling indicate overall robustness of EV extraction and RNA sequencing. Although this study offers potential mechanistic insights into vicadrostat and empagliflozin treatments, the newly identified biomarkers are not intended to monitor treatment response without further research and validation. Finally, the 8-week run-in period for empagliflozin randomization might have affected uEV miRNAs. Overall, these limitations resulted in a rather weak correlation between the identified miRNAs and UACR. Nevertheless, the robust expression of miR-142-5p, miR-192-5p, and miR-194-5p, with low 95% confidence intervals, offer potential as mechanistic biomarkers for future studies.

In conclusion, our findings demonstrate that uEV miRNAs were responsive to a CKD treatment intervention and reflected distinct mechanistic potential for the effects of vicadrostat, empagliflozin, or their combination in a broad range of kidney cell types. The robust and sustained modulation of miRNA profiles, especially under vicadrostat plus empagliflozin treatment, highlights their potential use as biomarkers for disease activity and treatment response in CKD.

## 4. Materials and Methods

### 4.1. Clinical Trial

Participants (male and female) with CKD, with or without T2D, were initially randomized to receive empagliflozin 10 mg once daily or a matched placebo, alongside RAS inhibition, for an 8-week run-in period [22]. Subsequently, participants underwent a second randomization baseline to receive vicadrostat (3 mg, 10 mg, or 20 mg once daily) or a matched placebo for 14 weeks, followed by a 4-week follow-up. The primary outcome was the change in UACR in the first morning void urine (FMV) from baseline to week 14. The main secondary outcome was a decrease in UACRFMV of ≥30% from baseline after 14 weeks. Additional outcomes included changes in estimated glomerular filtration rate (eGFR), blood pressure, and serum potassium from baseline to week 14. Of the 586 participants randomized at baseline, 452 participants completed the study [8]. Urinary samples available from 435 patients were included in the miRNA assessment, from which sets (minimum at baseline and end of treatment) were available for 412 patients.

### 4.2. Urinary EV Small RNA Sequencing

Spot urine samples (9 mL) were collected and stored immediately at −20 °C. Urine samples were centrifuged for 10 min at 16,000× *g* and 4 °C before RNA isolation. No freeze–thaw cycles were included. uEV miRNAs were isolated using the exoRNeasy Serum/Plasma Maxi Kit (Qiagen, Hilden, Germany). Characterization of EVs was previously described [44,45]. miRNA libraries were prepared using the QIAseq miRNA Library Kit (Qiagen, Hilden, Germany) following the manufacturer’s protocol, starting with ∼1 ng of input RNA for each sample. Quality control and concentrations of individual libraries were assessed using a Bioanalyzer 2100 Instrument and High Sensitivity DNA Kit (Agilent Technologies, Santa Clara, CA, USA). A miRNA-sized library is approximately 200 bp, and libraries were diluted to 4 nM. Small RNA sequencing was performed on the Illumina NovaSeq 6000 platform (Illumina Inc., San Diego, CA, USA) as 85 bp cycle single-end read. Ten million reads per sample were used as the cut-off.

### 4.3. Bioinfomatic Analysis

Exploratory analyses of changes in miRNA expression levels over time were performed to identify differentially expressed miRNAs (Figure S1). Preprocessing of the data (quality check, adapter trimming, reads alignment, miRNA reads quantification, quality control metrics) was performed using nf-core/smrnaseq: v2.3.1-g5901bea from Nextflow: 23.10.1. Exploratory analyses of changes in miRNA expression levels over time were performed to identify differentially expressed miRNAs. This analysis was conducted using the limma package [46]. Briefly, only miRNAs with counts per million (CPM) >= 1 in at least half the samples in at least one subgroup were included in the analysis. Data were normalized using the TMM method described by Robinson and Oshlack and voom-transformed [47]. To account for correlation between subjects, the duplicateCorrelation function was used with subject as a blocking factor, a linear model was fit using the lmFit-function and, finally, moderated t-statistics were computed to derive the log2 fold change. miRNAs were reported as differentially expressed if the fold-change was ≥1.5 and *p* ≤ 0.01.

### 4.4. Statistical Analysis

Correlation analysis was conducted to examine the relationship between miRNA expression and the UACR percentage change from baseline to end of treatment (EoT). Statistical analysis was performed using Spearman’s rank correlation. MiRNA expression at baseline was compared across the disease subgroups: diabetic kidney disease (type 2 diabetes), glomerular disease (FSGS, IgAN, MN), and hypertensive disease was performed using the Wilcoxon rank-sum test. Group comparisons over time were performed using a paired Wilcoxon rank-sum test. The enrichment analysis was performed using this R package:clusterProfiler 4.0: A universal enrichment tool for interpreting omics data: The Innovation. Predicted mRNA targets from the miRNA candidates were obtained from MSigDB (Molecular Signature Database) using the filters msigdbr (species = “human”, category = “C3”, subcategory = “MIRDB”) [48,49]. Universe is defined as a list of all predicted mRNA targets for all annotated miRNAs in the MSigDB. Cell type-specific expression was evaluated using publicly available data for human conditionally immortalized podocytes, glomerular endothelial cells, mesangial cells, and proximal tubular cells (PTCs) (PRJNA905899) [14].

## Figures and Tables

**Table 1:** Baseline and demographic and clinical characteristics of study participants.

	Vica_high_	Vica_low_	Empa	Vica_high_ + Empa	Vica_low_ + Empa	Placebo
	*n* = 99	*n* = 59	*n* = 54	*n* = 101	*n* = 62	*n* = 62
Gender						
Female	35 (35%)	15 (25%)	23 (43%)	32 (32%)	21 (34%)	17 (27%)
Male	64 (65%)	44 (75%)	31 (57%)	69 (68%)	41 (66%)	45 (73%)
Age (years)	65 (10)	65 (11)	65 (10)	61 (13)	66 (11)	62 (12)
Ethnicity/Race						
Asian	25 (25%)	20 (34%)	13 (24%)	38 (38%)	14 (23%)	12 (19%)
Black or African American	14 (14%)	3 (5.1%)	9 (17%)	8 (7.9%)	6 (9.7%)	9 (15%)
White Other/Mixed	56 (57%) 4 (4.0%)	33 (56%) 3 (5.1%)	31 (57%) 1 (1.9%)	52 (51%) 3 (3.0%)	41 (66%) 1 (1.6%)	38 (61%) 3 (4.8%)
Diabetes						
Yes	64 (65%)	41 (69%)	38 (70%)	65 (64%)	54 (87%)	42 (68%)
No	35 (35%)	18 (31%)	16 (30%)	36 (36%)	8 (13%)	20 (32%)
BMI (kg/m^2^)	29.9 (4.7)	30.0 (4.9)	29.5 (5.5)	29.4 (5.5)	30.1 (5.7)	30.5 (6.2)
eGFR (mL/min/1.73 m^2^)	55 (17)	53 (16)	49 (18)	52 (19)	50 (17)	56 (19)
UACR (mg/g)	615 (593)	848 (1,350)	690 (810)	759 (897)	770 (843)	715 (747)
SBP (mmHg)	137 (16)	135 (20)	133 (13)	134 (15)	135 (16)	134 (16)
DBP (mmHg)	77 (9)	78 (9)	77 (9)	77 (9)	76 (9)	81 (10)
Serum potassium (mmol/L)	4.29 (0.51)	4.24 (0.39)	4.23 (0.41)	4.28 (0.36)	4.35 (0.40)	4.29 (0.39)
Serum aldosterone (pmol/L) Participants with	174 (236)	195 (181)	160 (132)	148 (133)	163 (125)	175 (134)
<30% reduction	49 (49%)	40 (68%)	40 (68%)	38 (38%)	40 (65%)	53 (85%)
≥30% reduction	49 (49%)	19 (32%)	19 (32%)	63 (62%)	21 (34%)	9 (15%)
Unknown	1 (1.0%)	0 (0%)	0 (0%)	0 (0%)	1 (1.6%)	0 (0%)

*n* (%); Mean (SD); Vica, Vicadrostat; Empa, Empagliflozin; Vica_high_, Vica 10 mg + Vica 20 mg; Vica_low_, Vica 3 mg, BMI, body mass index; SBP, systolic blood pressure; DBP, diastolic blood pressure.

**Table 2:** Changes in uEV miRNAs and correlations with UACR by Vica_high_ or Vica_high_Empa treatment.

miRNA	EoT/Baseline (Vica_high_/Empa)	*p*-Value	EoT/Baseline (Vica_high_)	*p*-Value	Correlation with UACR (*p*-Value)
miR-142-5p	−1.83	0.048	−2.92	0.002	0.26 (0.001)
miR-192-5p	−1.97	0.001	−1.03	0.906	0.28 (<0.001)
miR-194-5p	−1.71	0.009	−1.34	0.246	0.24 (0.002)
miR-27a-5p	−1.76	0.003	−1.37	0.134	0.21 (0.018)
miR-381-3p	−1.54	0.001	1.08	0.551	0.25 (0.027)
miR-192-3p	−1.70	0.000	−1.07	0.647	0.23 (0.031)
miR-199b-3p	−1.58	0.008	−1.39	0.076	0.18 (0.044)
miR-513a-5p	−1.93	0.000	1.25	0.283	0.17 (0.038)
miR-6882-5p	−1.82	0.000	1.09	0.619	0.27 (0.014)

Fold-changes, *p*-values, and correlation coefficients are summarized. EoT, end of treatment; Vica, vicadrostat; Empa, empagliflozin; Vica_high_, Vica 10 mg + Vica 20 mg.

**Table 3:** Potential miRNA associations with mechanisms of kidney disease.

miRNA	Functions	References
miR-192-5p	Key role in transforming growth factor beta (TGFβ) signaling pathway. Reduced expression can be linked to reduced extracellular matrix proliferation and attenuated epithelial–mesenchymal transformation. Increased expression in patients with albuminuria.	[23,24,25]
miR-194-5p	Involved in TGFβ signaling pathway; reduced expression in patients with albuminuria.	[26]
miR-27a-5p	Increased miR-27a-5p in the pancreatic islets of genetic and dietary mouse models of obesity is mainly derived from visceral adipocyte-secreted EVs and serves as a pathogenic factor driving β-cell insulin secretion injury; involved in regulation of nuclear factor kappa B (NF-kB) signaling.	[27,28]
miR-381-3p	Functions as a dual suppressor of apoptosis and necroptosis and promotes proliferation of kidney cancer cells.	[29]
miR-192-3p	Increased level in urinary sediment obtained from membranous nephropathy compared to healthy controls by participating in inflammation and apoptosis.	[30]
miR-199b-3p	Significantly upregulated in ADPKD (Autosomal dominant polycystic kidney disease) patient urine extracellular vesicles.	[31]
miR-513a-5p	CircRTN4 (circular RNA derived from exon 4 and 5 of the Reticulon 4 (RTN4) mRNA) exacerbates mesangial cell dysfunction by activating the miR-513a-5p/FN (fibronectin) axis in lupus nephritis.	[32]
miR-6882-5p	Unknown.	
miR-142-5p	Increased expression in kidney fibrosis (results obtained from a meta-analysis). miR-142-5p is regulated by IL-4 and IL-13 and controls profibrogenic macrophage program; miR-142a-5p overexpression in activated lymphocytes shifts the pattern of T cell differentiation towards Th1 cells.	[33,34,35]

## Data Availability

To ensure the independent interpretation of clinical study results and enable authors to fulfill their role and obligations under the International Committee of Medical Journal Editors criteria, Boehringer Ingelheim grants all external authors access to relevant clinical study data. In adherence with the Boehringer Ingelheim policy on transparency and publication of clinical study data, scientific and medical researchers can request access to clinical study data, typically, 1 year after the approval has been granted by major regulatory authorities or after termination of the development program. Researchers should use the https://vivli.org/ (accessed on 15 September 2025) link to request access to study data and visit www.mystudywindow.com/msw/datasharing (accessed on 15 September 2025) for further information.

## Supplementary Materials

The following supporting information can be downloaded at https://www.mdpi.com/article/10.3390/ijms262210810/s1.
