# Supplementary material for: Urine Extracellular Vesicle miRNA Changes Induced by Vicadrostat with/Without Empagliflozin in Patients with Chronic Kidney Disease
Source: Int J Mol Sci. 2025 Nov 7;26(22):10810. doi: 10.3390/ijms262210810 (PMC12652424; doi:10.3390/ijms262210810)
Supplement: Supplementary file 1 [file ijms-26-10810-s001.zip › ijms-3886617-supplementary.pdf]

Supplementary Material

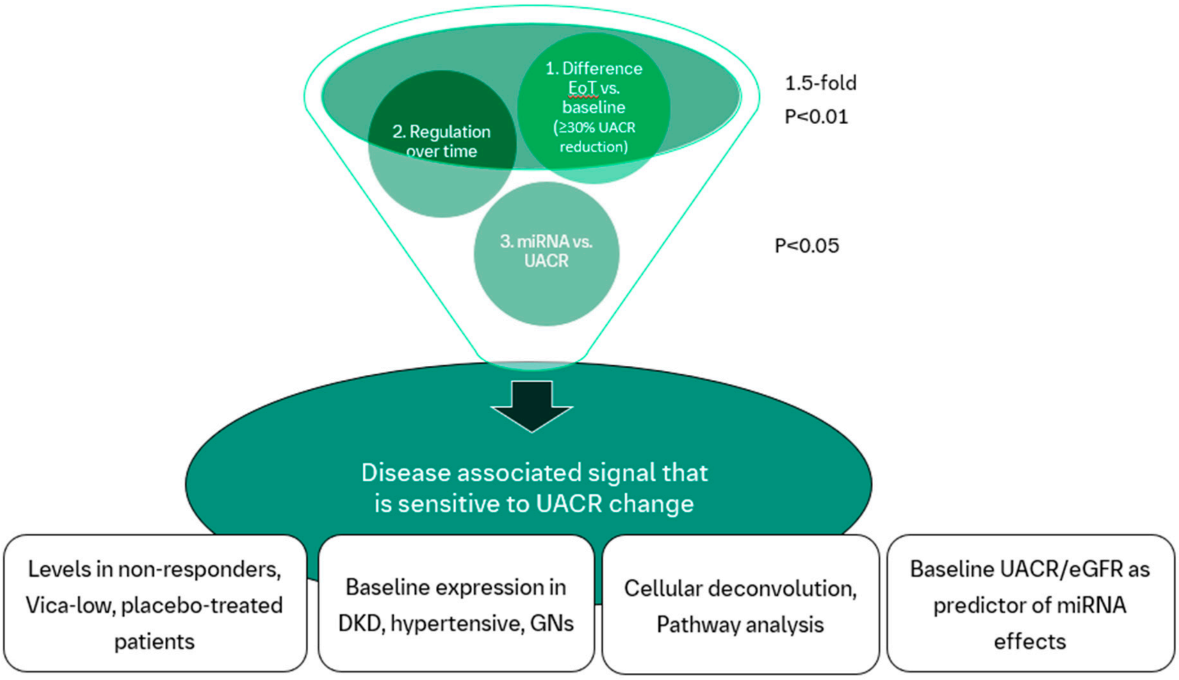

Figure S1. Experimental design to identify outcome related effects of Vicadrostat. Participants according to ≥30% change in UACR were pooled for 10 mg and 20 mg Vica +/- Empa groups- Vica high =10+20 mg; Vica low = 3 mg

Table S1. Outcome related uEV miRNAs modulated by Vica or VicaEmpa treatment associated with UACR changes less than 30%

miRNA	EoT/Baseline ($Vica_{high}$)	P-value	EoT/Baseline ($Vica_{high}$+Empa)	P-value	Treatment
miR-142-5p	-1.02	0.962	1.53	0.266	Vica
miR-192-5p	-1.08	0.762	-1.04	0.885	Vica+Empa
miR-194-5p	-1.09	0.715	1.19	0.508	Vica+Empa
miR-27a-5p	-1.3	0.221	1.08	0.766	Vica+Empa
miR-381-3p	-1.07	0.595	1.12	0.506	Vica+Empa
miR-192-3p	-1.46	0.01	1.02	0.91	Vica+Empa
miR-199b-3p	-1.34	0.115	1.17	0.474	Vica+Empa
miR-513a-5p	-1.05	0.808	1.09	0.714	Vica+Empa
miR-6882-5p	-1.28	0.143	1.04	0.83	Vica+Empa

n-$Vica_{high}$ =49; n-$Vica_{high}$ + Empa=38. Fold-changes, P-values and correlation coefficients are summarized. EoT, end of treatment; Vica, Vicadrostat; Empa, Empagliflozin; $Vica_{high}$,Vica 10 mg + Vica 20 mg

Table S2. Outcome related uEV miRNAs modulated by vicadrostat or vicadrostat plus empagliflozin treatment associated with UACR in placebo patients with ≥30% UACR reductions (n=9) and participants responding to vicadrostat low dose.

miRNA	EoT/Baseline	P-value	EoT/Baseline (Placebo)	P-value	Treatment
miR-142-5p	-1.06	0.914	1.2	0.84	Vica
miR-192-5p	-1.23	0.553	-1.84	0.306	Vica+Empa
miR-194-5p	-1.56	0.214	-1.25	0.715	Vica+Empa
miR-27a-5p	-1.01	0.964	1.15	0.789	Vica+Empa
miR-381-3p	-1.25	0.323	1.3	0.409	Vica+Empa
miR-192-3p	-1.25	0.392	-1.12	0.756	Vica+Empa
miR-199b-3p	-1.22	0.505	1.37	0.459	Vica+Empa
miR-513a-5p	-1.3	0.406	-1.84	0.214	Vica+Empa
miR-6882-5p	1.06	0.828	1.15	0.725	Vica+Empa

n-$Vica_{low}$=19; n-$Vica_{low}$+Empa = 20. Fold-changes, P-values and correlation coefficients are summarized. EoT, end of treatment; Vica, Vicadrostat; Empa, Empagliflozin; $Vica_{low}$=3mg Vica

Table S3. Outcome related uEV miRNAs modulated by vicadrostat or vicadrostat plus empagliflozin treatment associated with UACR in participants ≥ 30% UACR reduction with or without type II diabetes.

miRNA	EoT/Baseline (+diabetes)	P-value	EoT/Baseline (-diabetes)	P-value	Treatment
miR-142-5p	-2.25	0.065	-4.96	0.005	Vica
miR-192-5p	-1.92	0.005	-1.87	0.07	Vica+Empa
miR-194-5p	-1.71	0.027	-1.47	0.271	Vica+Empa
miR-27a-5p	-1.87	0.005	-1.2	0.596	Vica+Empa
miR-381-3p	-1.45	0.016	-1.46	0.121	Vica+Empa
miR-192-3p	-1.69	0.003	-1.41	0.186	Vica+Empa
miR-199b-3p	-1.51	0.043	-1.54	0.149	Vica+Empa
miR-513a-5p	-2.03	0.001	-1.38	0.312	Vica+Empa
miR-6882-5p	-1.95	0.001	-1.84	0.035	Vica+Empa

Fold-changes, P-values and correlation coefficients are summarized. EoT, end of treatment; Vica, Vicadrostat; Empa, Empagliflozin

Table S4. Correlation between baseline UACR and eGFR and % changes in uEV miRNA level (EoT vs. baseline).

miRNA	UACR	eGFR	Treatment
miR-142-5p	0.14 (0.085)	0.05 (0.509)	Vica
miR-192-5p	-0.04 (0.579)	-0.13 (0.081)	Vica + Empa
miR-194-5p	0.04 (0.642)	-0.05 (0.491)	Vica + Empa
miR-27a-5p	0.11 (0.232)	-0.02 (0.786)	Vica + Empa
miR-381-3p	<0.001 (0.994)	0.05 (0.665)	Vica + Empa
miR-192-3p	0.01 (0.356)	-0.19 (0.069)	Vica + Empa
miR-199b-3p	0.12 (0.162)	-0.15 (0.096)	Vica + Empa
miR-513a-5p	-0.08 (0.305)	0.05 (0.512)	Vica + Empa
miR-6882-5p	0.04 (0.75)	-0.01 (0.391)	Vica + Empa

Correlation coefficients and (P-values) are indicated

Table S5. Correlation between baseline uEV miRNA level and % changes in UACR and eGFR (EoT vs. baseline).

miRNA	UACR	eGFR	Treatment
miR-142-5p	-0.14<br>(0.066)	-0.12<br>(0.11)	Vica
miR-192-5p	-0.05<br>(0.466)	-0.02<br>(0.749)	Vica + Empa
miR-194-5p	-0.04<br>(0.613)	-0.01<br>(0.932)	Vica + Empa
miR-27a-5p	0.08<br>(0.349)	0.02<br>(0.829)	Vica + Empa
miR-381-3p	-0.08<br>(0.448)	0.2<br>(0.068)	Vica + Empa
miR-192-3p	-0.02<br>(0.854)	-0.03<br>(0.802)	Vica + Empa
miR-199b-3p	-0.01<br>(0.226)	-0.03<br>(0.763)	Vica + Empa
miR-513a-5p	-0.18<br>(0.018)	-0.03<br>(0.677)	Vica + Empa
miR-6882-5p	0.02<br>(0.868)	0.01<br>(0.924)	Vica + Empa

Correlation coefficients and (P-values) are indicated

Supplementary Table S6. Ethical Approval.

IRB/IEC	Protocol version approved	Date of approval	Approval Number
Fundación de Estudios Farmacológicos y de Medicamentos (FEFyM), Comité Independiente de Etica para Ensayos en Farmacolgia Clinica, 774 Pte JE Uriburu, Caba, C1027AAP, Argentina	1.0	9 November 2021	PR-0000002119
	2.0	15 December 2021	EN-0000002664
	3.0	22 June 2022	EN-0000002838
	4.0	26 October 2022	EN-0000002951
Comité de Ética en Investigación, Centro de Investigaciones Médicas Mar del Plata, Av. Colón 3083, Mar del Plata, Buenos Aires, B7600FYK, Argentina	1.0	11 November 2021	N/A
	2.0	22 December 2021	N/A
	3.0	14 July 2022	N/A
	4.0	12 October 2022	N/A
Comité de Ética en Investigación Clínica (CEIC), Paraná 755, Piso 6 "A" y "B", Ciudad Autónoma de Buenos Aires, C1017AAO, Argentina	1.0	14 October 2021	5876
	2.0	30 January 2022	1814/106/2021
	3.0	7 July 2022	1814/106/2021
	4.0	30 October 2022	1814/106/2021
Comité de Ética y Revisión Institucional del Centro Médico Famyl CEyRI, Lebensohn 31, Junín, Buenos Aires, B6000BHA, Argentina	1.0	10 November 2021	N/A
	2.0	24 January 2022	N/A
	3.0	26 July 2022	N/A
	4.0	21 November 2022	N/A
Comité de Bioética Cimel, Tucumán 1314, Lanús Este, Buenos Aires, B1824KAJ, Argentina	2.0	19 January 2022	CBC-01-2022
	3.0	20 July 2022	N/A
	4.0	16 November 2022	N/A
Comité de Ética Saavedra, Ruiz Luis Huidobro 4693, Caba, Buenos Aires, C1430CKE, Argentina	2.0	8 April 2022	6347
	3.0	15 July 2022	N/A
	4.0	4 November 2022	N/A
	3.0	25 July 2022	4564

Instituto Investigaciones Clínicas Córdoba, Deán Funes 1161, Córdoba, X5000AAW, Argentina	4.0	28 November 2022	9096
Comité Iniciativa y Reflexión Bioética Rosario, Rioja 2926, Rosario, Santa Fe, S20002OJP, Argentina	3.0	12 July 2022	N/A
	4.0	20 October 2022	N/A
St Vincent's Hospital Melbourne Human Research Ethics Committee, 41 Victoria Parade, Fitzroy, VIC 3065, Australia	2.0	19 January 2022	HREC Ref: HREC 291/21
	3.0	17 June 2022	HREC Ref: HREC 291/21
	4.0	9 September 2022	HREC Ref: HREC 291/21
Ethisch Comité Onze-Lieve-Vrouwziekenhuis, Moorselbaan 164, Aalst 9300, Belgium	1.0	13 December 2021	2021/106
	2.0	27 February 2022	2021/106
	3.0	31 May 2022	2021/106
	4.0	3 October 2022	2021/106
Ethics Committee for Clinical Trials, 8 Damyan Gruev str., Sofia 1303, Bulgaria	2.0	20 April 2022	EKKI/CT-0401
	3.0	16 June 2022	EKKI/CT-0596
	4.0	26 October 2022	EKKI/CT-1016
Comitê de Ética em Pesquisa, Centro Universitário FMABC/SP, Av. Lauro Gomes 2000, Vila Sacadura Cabral, Santo André, Estado de São Paulo, 09060-870, Brazil	2.0	25 February 2022	5.263.790
	3.0	9 August 2022	5.571.426
	4.0	31 January 2023	5.870.502
Comitê de Ética em Pesquisa, Hospital Universitário João de Barros Barreto (UFPA), Rua dos Mundurucus 4487, Guamá, Belém, Estado de Pará, 66073-000, Brazil	2.0	4 July 2022	5.507.523
	3.0	7 July 2023	6.169.361
Comitê de Ética em Pesquisa em Seres Humanos da Faculdade Evangélica Mackenzie do Paraná, Rua Padre Anchieta 2770, Bigorrilho, Curitiba, Estado de Paraná, 80730-000, Brazil	2.0	12 May 2022	5.404.046
	3.0	22 September 2022	5.659.503
Comitê de Ética em Pesquisa da Faculdade de Medicina de Botucatu, Universidad Estadual Paulista (UNESP), Chácara Butignoli s/n, Distrito de Rubião Junior, Botucatu, Estado de São Paulo, 18618-970, Brazil	2.0	8 June 2022	5.456.022
	3.0	11 November 2022	5.753.836
	4.0	23 March 2023	5.960.632

Comitê de Ética e Pesquisa em Seres Humanos do Hospital Pro Cardíaco, Rua Voluntários da Pátria 435, Botafogo, Rio de Janeiro, Estado de São Paulo, 22270-005, Brazil	2.0	5 October 2022	5.456.022
Comitê de Ética em Pesquisa do Hospital de Clínicas de Porto Alegre (HCPA), Av. Protásio Alves 211, Bairro Rio Branco, Porto Alegre, Estado de Rio Grande do Sul, 90410-000, Brazil	2.0	06 April 2022	5.334.916
	3.0	14 September 2022	5.642.215
	4.0	08 March 2023	5.932.204
Comitê de Ética em Pesquisa do Instituto de Saúde e Bem Estar da Mulher (ISBEM), Rua Peixoto Gomide 515, Bairro Jardim Paulista, São Paulo, Estado de São Paulo, 01409-001, Brazil	2.0	25 April 2022	5.367.393
	3.0	1 November 2022	5.733.462
	4.0	28 March 2023	5.967.700
Comitê de Ética em Pesquisa do Instituto de Pesquisa Clínica de Campinas, Grupo Investiga, Avenida Romeu Tórtima 739, Campinas, Estado de São Paulo, 13084-791, Brazil	2.0	29 April 2022	5.378.443
	3.0	26 September 2022	5.664.423
	4.0	21 March 2023	5.956.229
Comitê de Ética em Pesquisa do Hospital Municipal São José (HMSJ), Joinville (SC), Avenida Getúlio Vargas 238, Bairro Centro, Joinville, Estado de Santa Catarina, 89202-000, Brazil	2.0	09 May 2022	5.397.745
	3.0	12 September 2022	5.637.205
	4.0	14 August 2023	6.238.950
Comitê de Ética em Pesquisa do Hospital Beneficência Portuguesa de São Paulo, Rua Maestro Cardim 769, Bairro Liberdade, São Paulo, Estado de São Paulo, 01323-900, Brazil	2.0	31 May 2022	5.440.431
Invitare Pesquisa Clínica Auditoria e Consultoria, Rua Caramuru 417, Chácara Inglesa, São Paulo, Estado de São Paulo, 04138-001, Brazil	2.0	10 May 2022	5.400.243
	3.0	13 September 2022	5.639.237
	4.0	28 February 2023	5.917.345
Advarra, 372 Hollandview Trail, Aurora, Ontario, L4G 0A5, Canada	2.0	5 January 2022	SSU00168216
	3.0	20 April 2022	N/A
	4.0	25 August 2022	N/A
Advarra, 372 Hollandview Trail, Aurora, Ontario, L4G 0A5, Canada	2.0	4 January 2022	SSU00172620, SSU00172663
	3.0	20 April 2022	N/A

	4.0	25-Aug-2022	N/A
Advarra, 372 Hollandview Trail, Aurora, Ontario, L4G 0A5, Canada	2.0	21 January 2022	SSU00174402
	3.0	20 April 2022	N/A
	4.0	25 August 2022	N/A
Advarra, 372 Hollandview Trail, Aurora, Ontario, L4G 0A5, Canada	3.0	12 August 2022	SSU00193584
	4.0	25 August 2022	N/A
Advarra, 372 Hollandview Trail, Aurora, Ontario, L4G 0A5, Canada	4.0	7 September 2022	SSU00194736
Health Research Ethics Board of Alberta (HEREBA) Clinical Trials Committee, 10104 103 Ave NW #1500, Edmonton, AB T5J 0H8, Canada	2.0	1 June 2022	HREBA.CTC-22-0009
	3.0	1 June 2022	HREBA.CTC-22-0009_MOD1
	4.0	1 June 2022	HREBA.CTC-22-0009_MOD4
University Health Network Research Ethics Board, 700 University Ave, Toronto,<br>Ontario, M5G 1Z5, Canada	2.0	9 September 2022	21-6017
	3.0	9 November 2022	21-6017
Kantonale Ethikkommission Bern, Murtenstrasse 31, 3010 Bern, Switzerland	3.0	11 November 2022	2022-00226
	4.0	6 January 2023	2022-00226
Guangdong Provincial People's Hospital Ethics Review Committee, Guangdong Provincial People's Hospital, Donghua South Road, Yuexiu District, Guangzhou, Guangdong Province, 510080, China	2.0	15 December 2021	YW-2021-109-03
	3.0	3 August 2022	YW-2021-109-05
	4.0	5 January 2023	YW-2021-109-07
Shanghai Fifth People's Hospital affiliated to Fudan University, Heqing Road, Minhang District, Shanghai, Shanghai Municipality, 200240, China	2.0	18 February 2022	(2022）No.（045)
	3.0	15 September 2022	(2022）No.（045X2)
	4.0	28 April 2023	(2022）No.（045X3)
The First People's Hospital of Nanning, 89 Qixing Road, Nanning, Guangxi Zhuang, 530000, China	2.0	23 February 2022	2022-No.-026
	3.0	26 August 2022	2022-No.-103

	4.0	8 March 2023	2023-No.-023
Fudan University, Shanghai Huashan Hospital, 12 Urumqi Road, Shanghai Municipality, 200040, China	2.0	5 August 2022	2022 Temporary Examination No. （698
	3.0	12 October 2022	2022 Temporary Examination No. （698）Amendment 1
	4.0	16 March 2023	2022 Temporary Examination No. （698） Amendment 2
Zhejiang Province People's Hospital, 158 Shangtang Road, Hangzhou, 310014, China	2.0	14 March 2022	Zhejiang Medical Ethics Review 2022 Drug No. (008)
	3.0	12 September 2022	Zhejiang Medical Ethics Review 2022 Drug No. (008)
	4.0	10 March 2023	Zhejiang Medical Ethics Review 2022 Drug No. (008)
The First Affiliated Hospital, Sun Yat-Sen University, 58 Zhongshan Er Road, Guangzhou, Guangdong Province, 510080, China	2.0	30 June 2022	2022-087-01
	3.0	28 February 2023	2022-087-02
Eticka komise Vseobecne fakultni nemocnice v Praze, Na Bojisti 1, 128 08 Praha 2, Czech Republic	3.0	21 April 2022	250/21 S-MEK
	4.0	15 September 2022	1271/22 A,D
Eticka komise, Nemocnice Slany, Politickych veznu 576, 274 01 Slany, Czech Republic	2.0	31 December 2021	31122021
	3.0	29 April 2022	29042022
	4.0	30 September 2022	30092022
Eticka komise, Krajska nemocnice Liberec, Husova 357/10, 460 63 Liberec, Czech Republic	2.0	27 April 2022	EK/29/2022
	3.0	27 April 2022	EK/29/2022
	4.0	21 September 2022	EK/66/2022
Ethik-Kommission bei der Medizinischen, Fakultät der Universität Würzburg, Institut für Pharmakologie und Toxikologie, Versbacher Str. 9, 97078 Würzburg, Germany	2.0	9 February 2022	311/21_ff
	3.0	6 May 2022	311/21_ff
	4.0	1 August 2023	311/21_ff
Hospital Universitario Puerta de Hierro de Majadahonda, C. Joaquin Rodrigo 2, CP 28222, Madrid, Spain	2.0	28 March 2022	07/812264.9/22
	3.0	19 July 2022	N/A
	2.0	19 January 2022	Dnro ETMK 105/2021

Varsinais-Suomen sairaanhoitopiirin eettinen toimikunta, Kiinamyllynkatu 4-8, PL 52 Turku, FI-20521, Finland	3.0	20 May 2022	Dnro ETMK: 105/1800/2021
	4.0	23 November 2022	Dnro ETMK: 105/1800/2021
National Ethics Committee, 284 Mesogion Avenue, Holargos 15562, Greece	2.0	14 April 2022	11646/2022
	3.0	21 July 2022	65797/2022
	4.0	20 October 2022	100444/2022
Institutional Review Board of the University of Hong Kong, Hospital Authority Hong Kong West Cluster, Queen Mary Hospital, Hong Kong	2.0	19 January 2022	UW 22-032
	3.0	26 May 2022	UW 22-032
	4.0	03 November 2022	UW 22-032
Joint CUHK-NTEC Clinical Research Ethics Committee, Lui Che Woo Clinical Sciences Building, Prince of Wales Hospital, Shatin, 999077, Hong Kong	2.0	31 March 2022	2021.738-T
	3.0	16 June 2022	2021.738-T
	4.0	21 November 2022	2021.738-T
Medical Research Council Ethics Committee for Clinical Pharmacology, Alkotmány street 25, Budapest 1054, Hungary	2.0	4 February 2022	OGYÉI/79148-6/2021, EC: IV/10195-0/2021-EKL
	3.0	25 May 2022	OGYÉI/26515-5/2022, EC: IV/3720-0/2022-EKL
	4.0	28 November 2022	OGYÉI/56997-4/2022, EC: BMEÜ/3653-0/2022-EKL
Institute Ethics Committee All India Institute of Medical Sciences, All India Institute of Medical Sciences, Ansari Nagar, New Delhi, 110029, India	2.0	11 February 2022	IEC- 22/14.01.2022, RP-12/2022, OP-15/04.02.2022
	3.0	20 July 2022	IEC- 22/14.01.2022, RP-12/2022, OP-15/04.02.2022,<br>OP-07/15.07.2022
	4.0	11 October 2022	IEC- 22/14.01.2022, OP-22/07.10.2022
Christian Medical College Ethics Committee Silver, Christian Medical College, Carman Block Vellore, Tamil Nadu, 32002, India	2.0	21 May 2022	14549
	3.0	27 July 2022	IRB-A10-29.06.2022
	4.0	19 October 2022	IRB-A19-28.09.2022
K R Hospital Mysore Medical College and Research Centre Institutional Ethics Committee, K R Hospital Mysore Medical College and Research Centre, Irwin Road, Mysore, Karnataka, 570001, India	2.0	21 March 2022	MMC EC 16/22
	3.0	21 June 2022	N/A
	4.0	10 April 2023	MME EC 34/23
Jaipur National University Institute for Medical Science & Research Centre Institutional Ethics Committee, Jaipur National University Institute for Medical Science & Research Centre, Jagatpura, Jaipur, Rajasthan, 302017, India	2.0	21 March 2022	JNUIMSRC/ IEC/2022/11
	3.0	20 May 2022	JNUIMSRC/ IEC/2022/81
	4.0	11 October 2022	JNUIMSRC/ IEC/2022/93

Galaxy Hospital Ethics Committee, Galaxy Life Care Services Private Limited, Dayal Enclave, Mahmoorganj, Varanasi, Uttar Pradesh, 221010, India	2.0	10 January 2022	N/A
	3.0	02 July 2022	N/A
	4.0	26 December 2022	N/A
Shree Giriraj Hospital Research Ethics Committee, 27-Navjyot Park, 150 Feet Ring Road, Rajkot, Gujarat, 360005, India	2.0	12 January 2021	N/A
	3.0	12 July 2022	N/A
	4.0	14 October 2022	N/A
Unity Hospital Ethics Committee, Opp. Raghuvir Business Empire, Aai Mate Road, Parvat Patiya, Surat, Gujarat, 395010, India	2.0	21 Dec 2021	N/A
	3.0	02 Jun 2022	N/A
Kingsway Hospitals Ethics Committee, Kingsway Hospitals, 44 Kingsway, Nagpur, Maharashtra, 440001, India	2.0	22 March 2022	N/A
	3.0	20 June 2022	N/A
	4.0	17 January 2023	N/A
SMS Medical College and Hospital, Jawahar Lal Nehru Marg, Ashok Nagar, Jaipur, Rajasthan, 302001, India	2.0	20 May 2022	N/A
	3.0	10 April 2023	N/A
	4.0	10 Apr 2023	N/A
Ganesh Shankar Vidyarthi Memorial Medical College, Swaroop Nagar, Kanpur, Uttar Pradesh, 208002, India	2.0	15 Dec 2021	EC/282/ Dec/2021
	3.0	24 Jun 2022	EC/191/June/2022
	4.0	19 Jun 2023	123/ EC/Jun/2023
Comitato Etico Aziende Sanitarie Locali (ASL) di Bergamo, Papa Giovanni xxiii Hospital, piazza OMS, 1, Bergamo, 24127, Italy	2.0	31 March 2022	N/A
	3.0	14 July 2022	N/A
Policlinico di Bari, Piazza Giulio Cesare 11, Bari, 70121, Italy	2.0	23 February 2022	N/A
	3.0	7 September 2022	N/A
	2.0	11 March 2022	N/A

Review Board of Human Rights and Ethics for Clinical Studies Institutional Review Board, 2-2-1, Kyobashi, Chuo-ku, Tokyo, Japan	3.0	24 June 2022	N/A
	4.0	28 October 2022	N/A
Review Board of Human Rights and Ethics for Clinical Studies Institutional Review Board, 2-2-1, Kyobashi, Chuo-ku, Tokyo, Japan	2.0	16 March 2022	N/A
	3.0	27 June 2022	N/A
	4.0	1 November 2022	N/A
Review Board of Tokyo University Hospital, Tokyo University Hospital, 7-3-1, Hongo Tokyo, Bunkyo-ku, 113-8655, Japan	2.0	22 April 2022	N/A
	3.0	28 June 2022	N/A
	4.0	1 November 2022	N/A
Review Board of Human Rights and Ethics for Clinical Studies Institutional Review Board, 2-2-1, Kyobashi, Chuo-ku, Tokyo, Japan	2.0	2 May 2022	N/A
	3.0	24 June 2022	N/A
	4.0	28 October 2022	N/A
Review Board of Tokyo Medical University Hachioji Medical Center, 1163 Tatemachi, Hachioji-shi, Tokyo 193-0998, Japan	3.0	26 September 2022	N/A
	4.0	28 October 2022	N/A
Review Board of Human Rights and Ethics for Clinical Studies Institutional Review Board<br>2-2-1, Kyobashi, Chuo-ku, Tokyo, Japan<br>(Site: JPN18)	4.0	11 November 2022	N/A
Review Board of Human Rights and Ethics for Clinical Studies Institutional Review Board<br>2-2-1, Kyobashi, Chuo-ku, Tokyo, Japan<br>(Site: JPN2)	2.0	14 January 2022	N/A
	3.0	24 June 2022	N/A
	4.0	28 October 2022	N/A
Review Board of Human Rights and Ethics for Clinical Studies Institutional Review Board<br>2-2-1, Kyobashi, Chuo-ku, Tokyo, Japan<br>(Site: JPN6)	2.0	14 January 2022	N/A
	3.0	27 June 2022	N/A
	4.0	31 October 2022	N/A
Severance Hospital, Institutional Review Board, 50-1 Yonsei-ro, Seodaemun-gu, Seoul 03722, Republic of Korea<br>(Site no.: KOR1)	Initial	22 November 2021	4-2021-1374
	2.0	27 December 2021	N/A
	3.0	22 April 2022	N/A
The Catholic University of Korea, Seoul St. Mary's Hospital Institutional Review Board 222 Banpo-daero,Seocho-gu, Seoul 06591, Republic of Korea<br>(Site no.: KOR2)	Initial	25 March 2022	KIRB-20220325-056
	3.0	15 June 2022	N/A
Seoul National University Hospital, Institutional Review Board, 101 Daehak-ro, Jongno-gu Seoul 03080, Republic of Korea	Initial	30 November 2021	IRB - 2109-147-002
	2.0	15 December 2022	N/A

(Site no.: KOR3)			
Inje University Ilsan Paik Hospital Institutional Review Board Juhwa-ro 170, Ilsanseo-gu Goyang 10380, Republic of Korea<br>(Site no.: KOR4)	Initial	2 December 2021	ISPAIK 2021-09-010-003
	2.0	7 January 2022	N/A
Korea University Ansan Hospital Institutional Review Board 123, Jeokgeum-ro, Danwon-gu Ansan 15355, Republic of Korea<br>(Site no.: KOR5)	3.0	20 June 2022	K2022--08530853-001001
Chungbuk National University Hospital, Institutional Review Board, Chungdae-ro 1, Seowon-Gu Cheongju 28644, Republic of Korea<br>(Site no.: KOR6)	3.0	12 May 2022	CBNUH 2022-04-026
The Catholic University of Korea, Eunpyeong St. Mary's Hospital 1021,Tongil Ro, Eunpyeong-gu Seoul 03312,<br>Republic of Korea<br>(Site no.: KOR7)	3.0	7 July 2022	PIRB-20220707-038
SMG-SNU Bramae Medical Center Institutional Review Board 20 Boramae-Ro 5 Gil, Dongjak-gu Seoul 07061, Republic of Korea<br>(Site no.: KOR8)	3.0	24 May 2022	20-202 2-60
Comité de Ética en Investigacion de Investigación biomédica para el Desarrollo de fármacos, Calle Volcán Popocatépetl No. 3352 Colonia Colli Urbano Zapopan Jalisco, Guadalajara CP, 45070, Mexico	1.0	28 October 2021	CEI-000001
	2.0	25 November 2021	CEI-000003
	3.0	25 July 2022	CEI-000005
	4.0	15 November 2022	CI-000008
Comite de Etica en Investigacion del Centro Integral en Reumatologia (CIRSA), Avenida La Paz N0. 1917 y 1919, Col. Americana, Guadalajara Jalisco; Guadalajara CP, 44160, Mexico	2.0	4 January 2022	N/A
	3.0	26 July 2022	N/A
	4.0	13 December 2022	N/A
Centenario Hospital Miguel Hidalgo<br>Col. La Estación, La Alameda Av. Gomez Morin S/N Aguascalientes; Aguascalientes, 20259, Mexico	3.0	21 September 2022	CI/082/022
	4.0	16 February 2023	CI/020/23
Hospital Universitario Dr Jose Eleuterio Gonzalez Comite de Etica en Investigacion / Comite de Investigacion. Col. Mitras Centro Avenida Francisco I. Madero y Av. Gonzalitos S/N Monterrey Nuevo León, Monterrey 64460, Mexico	2.0	18 February 2022	EN22-00002
	3.0	25 August 2022	N/A
	4.0	9 November 2022	N/A
Instituto Nacional de Ciencias Médicas y Nutricion Salvador Zubiran , Avenida Vasco de Quiroga, N° 15 Colonia Belisario	2.0	3 March 2022	298/2022<br>Reg 4047
	3.0	23 May 2022	791/2022

Domínguez Sección XVI Alcadía Tialpan, Ciudad de Mexico, CP 14080, Mexico	4.0	6 October 2022	1567/2022
Comité de Ética en Investigacion de Investigación biomédica para el Desarrollo de fármacos, Calle Volcán Popocatépetl No. 3352 Colonia Colli Urbano Zapopan Jalisco, Guadalajara CP, 45070, Mexico	1.0	8 November 2021	CEI-000001
	3.0	19 July 2022	CEI-000005
	4.0	8 November 2022	CEI-000006
Investigación Farmacológica y biofarmacéutica SAPI de CV periférico sur número 4927, arenal Tepepan, Tlalpan Ciudad de Mexico, 14610, Mexico	1.0	18 November 2021	CEI-211118-497-1
	2.0	13 January 2022	CEI-220113-006-1
Comité de Ética en Investigación del Hospital Hispano, S.A. de C.V. Pedro Moreno No. 934, Colonia Centro, Guadalajara, Jalisco. CP, 44100, México	2.0	20 May 2022	N/A
	3.0	27 June 2022	N/A
	4.0	28 October 2022	N/A
Hospital Hispano, S.A. de C.V. Zona Centro Sector Juárez Pedro Moreno #934, Guadalajara Jalisco, Guadalajara, 44100, Mexico	2.0	15 December 2021	N/A
	3.0	4 August 2022	N/A
	4.0	20 January 2023	CEI-000007
Comite de Etica en Investigacion Centro Especializado en Diabetes Obesidad y Prevencion de Enfermeda (CEDOPEC) Col. Reforma Social Calle 3 No. 7, Ciudad de Mexico, 11650, Mexico	2.0	13 December 2021	324-CI-12-21
	3.0	8 June 2022	127-CI-06-22
	4.0	07 November 2022	242-CI-11-22
Medical Research & Ethics Committee d/a Kompleks Institut Kesihatan Negara Blok A, No 1, Jalan Setia Murni U13/52 Seksyen U 13, Bandar Setia Alam Selangor Shah Alam, 40170, Malaysia	2.0	11 February 2022	(2)KKM/NIHSEC/ 61541
	3.0	08 June 2022	(4)KKM/NIHSEC/ 61541
Hospital University Kebangsaan Malaysia Centre for Research and Instrumentation Management, Research Ethics Committee 1st Floor, Clinical Block, Hospital Canselor, Tunku Mukhriz UKM Medical Centre, Jalan Yaacob Latif, Bandar Tun Razak, Kuala Lumpur, 56000 Malaysia	2.0	28 February 2022	UKM PPI/111/JEP-2022-006
	3.0	20 April 2022	N/A
	4.0	20 September 2022	N/A
Regional komite for med. og helsefaglig forskningsetikk REK Sør-Øst Postboks 1130 Blindern Gullhaugveien 1-3, 0484, Oslo, N-0318, Norway	2.0	7 February 2022	343117
	3.0	26 April 2022	343117
	4.0	8 September 2022	343117
Philippine Heart Center, Philippine Heart Center Institutional Ethics Review Board 8F Medical Arts building, East Avenue, Quezon, 1100, Philippines (Site no.: PHL11)	Initial	17 August 2022	L-E-CRD-CTR-2022-655
	4.0	17 October 2022	L-E-IRB-2022-436
	Initial	1 August 2022	1349/P/2022/159

University of the East-Ramon Magsaysay, Memorial Medical Center. Auroro Boulevard, Quezon City, 1113, Philippines (Site no.: PHL12)	4.0	9 December 2022	N/A
Institutional Review Board Chong Hua Hospital, Don Mariano Cui St., Corner J. Llorente St., Cebu City, Philippines 6000 (Site no.: PHL2)	Initial	16 September 2021	8121-08
	2.0	18 November 2021	N/A
	3.0	27 April 2022	N/A
	4.0	27 September 2022	N/A
University of the East-Ramon Magsaysay Memorial Center, 64 Aurora Boulevard, Barangay,Doña Imelda, Quezon City, 1113, Philippines	Initial	2 September 2021	1103/P/2021/183
	2.0	16 November 2021	N/A
	3.0	2 May 2022	N/A
	4.0	15 September 2022	N/A
The Medical City, Ortigas Avenue, Pasig City, Philippines (Site no.: PHL6)	Initial	16 November 2021	N/A
	2.0	14 December 2021	N/A
	3.0	17 May 2022	N/A
	4.0	27 October 2022	N/A
West Visayas State University Medical Center, E. Lopez St., Jaro Iloilo City, 5000, Philippines (Site no.: PHL7)	3.0	2 August 2022	N/A
	4.0	26 January 2023	N/A
Davao Doctors Hospital Research Ethics Committee, 118 E. Quirino Avenue, Davao City, 8000, Philippines (Site no.: PHL8)	Initial	18 August 2022	22-017-sis
	3.0	25 August 2022	N/A
	4.0	24 October 2022	N/A
Komisja Bioetyczna przy Bydgoskiej Izbie Lekarskiej Ul. Powstańców Warszawy 11 85-681 Bydgoszcz, Poland	2.0	20 February 2022	1/2022
	3.0	24 May 2022	38/2022
	4.0	20 September 2022	77/2022
CEIC Parque da Saúde de Lisboa, Av. do Brasil, 53 Pavilhao 17-A, Lisboa, 1749-004, Portugal	2.0	28 February 2022	MAP / MAP / OF / 2022 / 2693 / 20210899
	3.0	9 August 2022	JB / JB / OF / 2022 / 9878 / 20220534
Swedish Ethical Review Authority Etikprövningsmyndigheten Box 2110, 750 02, Uppsala, Sweden	2.0	11 January 2022	Dnr 2021-06495-01
	3.0	23 May 2022	Dnr 2022-02192-02
Hacettepe University Medical Faculty, Faculty of Medicine, Gastroenterology, Ic Hastaliklari A.B.D. Gastroenteroloji Bilim Dali, Ankara, 06100, Turkey	2.0	17 May 2022	2022/08-08
	3.0	13 June 2023	2023/11-08
	4.0	13 June 2023	2023/11-08
	1.0	21 July 2021	N/A

Alpha IRB, 1001 Avenida Pico, Suite C#497 San Clemente, CA 92673, USA	2.0	13 December 2021	N/A
	3.0	11 April 2022	N/A
	4.0	29 August 2022	N/A
Providence, 1801 Lind Ave SW, Renton, WA 98057, USA	1.0	11 November 2021	N/A
	2.0	10 February 2022	MOD2022000047
	3.0	21 April 2022	MOD2022000466
	4.0	1 September 2022	MOD2022001114
Cleveland Clinic, Institutional Review Board (FWA00005367), 9500 Euclid Avenue, Cleveland, OH 44195, USA	2.0	10 November 2022	N/A
	3.0	22 November 2022	N/A
	4.0	22 November 2022	N/A
Pharma Ethics, PO Box 786, Irene, 0062, 123 Amkor Road, Lyttelton Manor Ext 3, Centurion, Gauteng, Republic of South Africa	2.0	9 March 2022	N/A
	3.0	26 May 2022	220224507

IRB, Institutional Review Board; IEC, Independent Ethics Committee.
